# Statistical guidance for responsible data sharing: an overview

**DOI:** 10.1186/s12874-016-0168-5

**Published:** 2016-07-08

**Authors:** Christine Fletcher, Sally Hollis, Hans-Ulrich Burger, Christoph Gerlinger

**Affiliations:** Amgen Ltd., 240 Cambridge Science Park, Cambridge, CB4 0WD UK; AstraZeneca, Alderley Park, Cheshire, SK10 4TG UK; Centre for Biostatistics, Institute of Population Health, University of Manchester, Manchester Academic Health Science Centre, Oxford Road, Manchester, M13 9PL UK; Hoffman-La Roche, Grenzacherstrasse 124, 4070 Basel, Switzerland; Global Research and Development Statistics, Bayer Pharma AG, 13353 Berlin, Germany; Gynecology, Obstetrics and Reproductive Medicine, University Medical School of Saarland, 66421 Homburg, Saar Germany

Since at least 2004 there has been a focus on data sharing and clinical trial disclosure with the requirements for protocols to be registered in clinicaltrials.gov and for subsequent manuscripts of the study results to be accepted for publication by major journals [1]. However, sponsors of clinical trials have for many years been widely criticised for not adhering to these requirements and failing to disclose clinical trials in a timely fashion (e.g., www.alltrials.net].

In 2013 the European and US pharmaceutical trade bodies EPFIA/PhRMA published their *Principles for responsible sharing of clinical trial data* [2]. This was a voluntary but significant commitment to clinical trial transparency and it has led to a significant change in attitudes and behaviours towards sharing of clinical trial data. Many companies have developed processes for clinical trial data access schemes [3], aligned to the new EU Clinical Trials Regulation 536/2014 [4] and the European Medicines Agency’s transparency policies: Access to Documents (Policy 0043) [5] and Publications and Publication of clinical-trial data for medicinal products for human use (Policy 0070) [6]. Whilst there are common elements to these access schemes, there are many differences in terms of what existing clinical trial data are in scope for sharing between companies, ranging from all existing data are considered (no time limits) versus data available from 2015.

EFPIA recently reported that “According to the European Medicines Agency around 4000 trials are authorised each year across the European Economic Area” [7]. There is a wealth of clinical data being generated annually and the efforts by Industry and other data owners, coupled with changes in regulations, now enables medical researchers to seek access to a large number of sources of patient level data to support their medical research.

The European Federation of Statisticians in the Pharmaceutical Industry (EFSPI) issued a paper “*Position on Access to Clinical Trial Data”* in 2013 [8]. In this paper, EFSPI noted its support for responsible data sharing and highlighted a number of important aspects: ensuring that credible and robust research is conducted on any data shared; care is taken to avoid the misuse of data; there is confirmation that the original informed consent allows the data to be used in the proposed further research; and patient confidentiality is protected. However, EFSPI also recognized that further guidance and discussion on key principles and recommendations for efficient and effective sharing of individual patient data is needed. Therefore, EFSPI together with the UK based PSI (Statisticians in the Pharmaceutical Industry) initiated a working group to look deeper into the challenges of sharing individual patient data. The result of this effort has led to the development of four papers described below.

It is important to recognize that there can be significant challenges to working with existing patient level data sets, for example, data standards have evolved over time with different data owners following different definitions and formats. Different approaches for documenting and describing these standards have also been used. Whilst this is not unsurmountable, careful planning and handling of shared data is required to ensure data are correctly used in further analyses. Whilst it may be common in some research settings to share data between institutions, e.g., MRC have shared data between clinical trial units for some time [9] and processes and systems to do this effectively have thus been established, for other data owners sharing data is a relatively new concept.

Specific regulations and requirements exist to protect patient confidentiality of any data shared [10], and these must be followed to ensure data owners appropriately de-identify and anonymise data. There are significant concerns by some data holders regarding the potential for shared de-identified data to be re-identified despite having legally binding data sharing agreements in place to avoid this. To date, published case studies suggest this practice has generally focused on the linkage of patient information between large existing health related databases rather than clinical trial data sources. Given the use of specific informed consents used by patients in clinical trials that protect their confidentiality and identity, it is thus not surprising to see data owners being conservative in ensuring data are appropriately de-identified whilst maintaining as much data utility as possible. However, whilst there is alignment between some data owners on minimum expectations relating to de-identification, there is not yet full agreement on best practices.

Furthermore, there are important technical aspects to be considered relating to the re-analysis and/or supplemental analyses of shared data, such as what constitutes appropriate interpretation of results from re-analyses where different methods have been utilized; and potential over-interpretation, for example, of additional subgroups analysed. Finally, the increasing number of data sharing policies, processes and expertise required to manage requests for shared data is impacting the role of statisticians: not only does this bring challenges but it also provides a number of opportunities that statisticians can and should embrace. For example, further analysis of shared data will often result with new medical insights, and will often enable new clinical questions to be explored. Together this could influence and shape the roles and responsibilities of statisticians involved in designing, analyzing and reporting clinical trials in the future.

A substantial amount of individual patient data from pharmaceutical clinical development is now being made available through increased data sharing efforts described above. In order to make this effort really feasible and useful it is important for all data owners to adhere to data standards, for example CDISC, and to adhere to clinical trial principles defined in regulatory guidelines from ICH, for example Statistical Principles in Clinical Trials (ICH E9). In particular it will be essential for data owners to be transparent on how results were derived from study data such that other researchers have a chance to understand the original analyses to ensure appropriate interpretation of the results of their further analyses. This is not only a requirement for Industry but should also be followed by academia and other groups generating and maintaining clinical data. However, the analysis of shared data is complex as data standards and methods for analyses evolve over time, some inconsistencies are likely to occur when shared data are re-analysed. Where inconsistencies are found, understanding the reasons will be essential to maintain adequate data interpretation. For example, differences between results presented from a re-analysis when compared to results reported in a clinical study report (or in a publication of the study, or in the regulatory approval of the medicine) could be due to different data cuts, different analysis requirements, or different analysis populations. Often trying to rationalize these differences will be difficult as the available documentation may be limited in terms of describing all the steps of the analyses.

The EFSPI/PSI Data Sharing Working Group, which includes Industry and academic representatives, has developed a series of 4 papers that focus on these key topics: (1) (Sudlow et al: A primer for researchers working with patient level data sets, submitted), (2) (Tucker et al: Ensuring patient confidentiality when sharing patient-level data from clinical trials, submitted), (3) (Holis et al: Best practices for analysis of shared clinical trial data, submitted), and (4) (Manamley et al: Does data sharing change the role of statisticians?, submitted). All four papers discuss and debate a wide range of aspects for sharing data. This first paper discusses on the importance of researchers providing a through list of requirements seeking access to shared data to ensure that their proposed research can be fulfilled. The second paper provides an overview and recommendations for data owners on acceptable criteria for anonymization of shared data. While the mechanics of this will be most relevant to industry providers of shared data, it is important for users of these shared data to understand what measures may have been taken to anonymize data, and the potential impact of these on their analyses of the shared data. The third paper discusses the role of the analysis plan, and what are minimal criteria that need to be fulfilled in order to make a research plan meaningful. The analysis plan is considered not only a necessary condition to get access to individual patient data but an essential part of the research, and will impact the interpretation of results obtained. Finally, data sharing will very likely change the way industry and academia collaborate in the future. This may impact also on the abilities and proficiencies statisticians need to demonstrate to be successful in the future. This is discussed in the last paper.

There are some topics that have not been addressed in these papers. One topic concerns data sharing within industry. Whereas in academia there may be more open willingness to share data between institutions, due to issues relating to intellectual property rights, sharing data across companies is more challenging. To do this well, it would be beneficial if agreements could be achieved on principles for data sharing within Industry, for example, that any data shared is used for the stated scientific purposes only and it is not used to inform or fuel marketing strategies. There is clearly a scientific need for data sharing within industry, for example to improve study planning, and more accurately identify patients who have a high unmet medical need. Whilst statisticians can and should contribute to discussions relating to principles for sharing data between companies, they are unlikely to be the final decision makers. Another topic of interest concerns whether all data owners will eventually agree to a single framework and process for sharing data, if this is indeed even feasible. Whilst a number of technical solutions are in development, these are too new to explore at this time. In addition, there are an increasingly large number of consortiums being formed to develop shared data networks for specific disease areas and the impact this could have in future medical research. Finally, there is significant change in how clinical trial data are being captured with new digital technologies being utilized to collect source patient data. These new technologies will likely contribute to the ongoing transformation underway in how clinical trials are conducted, including how this data would become accessible for further research.

In summary, we can reasonable expect that broad and open data sharing as agreed today by pharmaceutical industry will have an impact on drug development and the information available to medical and patient communities. It is important that researchers in academia as well as in industry appreciate the new paradigm shift in data transparency and the new conditions this brings. This is particularly true for statisticians as they will remain deeply involved in data sharing, not just in providing data but also in synthesizing information from their own analyses and with those received from independent external researchers.

We acknowledge and thank those members of EFSPI and PSI who have contributed to the discussions and participating in the review of the data sharing publications.
